# Metal-organic frameworks (MOFs) and their derivatives as emerging biomaterials for the treatment of osteoarthritis

**DOI:** 10.3389/fphar.2024.1462368

**Published:** 2024-09-18

**Authors:** Yufu Liu, Hongwei Zhang, Tianyan Chen, Chang Xu, Xingfu Bao

**Affiliations:** Jilin Provincial Key Laboratory of Tooth Development and Bone Remodeling, School and Hospital of Stomatology, Jilin University, Changchun, China

**Keywords:** metal-organic frameworks, osteoarthritis, biomaterials, scaffold, bone tissue engineering

## Abstract

As a novel class of smart biomaterials with promising potentials, metal-organic frameworks (MOFs) are widely utilized in the field of biomedicine. Current researches indicate that the therapeutic strategies for osteoarthritis (OA) are highly limited to achieving symptom improvement and reducing both pain and inflammation. Together, the introduction of MOFs into the treatment of OA holds the potential to offer significant benefits. This is because MOFs not only have intrinsic biological activities, but also act as carriers to facilitate controlled drug delivery and prolong the duration in the management of OA. This paper presents a review of the recent studies that have explored the potential usage of MOFs as drugs or carriers in the treatment of OA, which also examines the progress of MOFs in tissue engineering for the treatment of OA. These studies are anticipated to not only enhance the comprehension of MOFs but also provide strong evidence in favor of their utilization in the treatment of OA.

## 1 Introduction

As a prevalent and destructive chronic disease, osteoarthritis (OA) has been considered as the primary cause of cartilage erosion. The etiology of OA mainly involves the following factors, such as age, weight, gender, genetic susceptibility, sports-related injury, and inflammation. It is well known that any joint in the body can be impacted along with the occurrence of OA, which can induce the patients to experience symptoms including pain, edema, stiffness, and restricted movement (Motta et al., 2023). According to recent studies, over 240 million worldwide individuals are believed to experience symptomatic OA, resulting in substantial physical and psychological strain on sufferers, especially among the elderly (Lv et al., 2024). More importantly, articular cartilage is composed of chondrocytes and an extracellular matrix (ECM) that surrounds the chondrocytes, which does not possess sufficient circulatory, neural, and lymphatic systems that are necessary for self-healing (Liu et al., 2021; Gao et al., 2022). Therefore, it has been proven to be a challenge that managing osteoarthritis through the regeneration of articular cartilage.

A series of medication including corticosteroids, non-steroidal anti-inflammatory drugs, and hyaluronic acid has been well developed to alleviate inflammation in OA. Compared with other alternative techniques, intra-articular injections are considered as the most efficacious method for treating OA (Richard et al., 2023; Guo et al., 2022). Although promising, it is still difficult to ensure the controlled release of drug and its long-term release effectiveness. Currently, scientists are exploring innovative approaches to improve the delivery of drugs with the assistance of nanotechnology. As well known, nanomaterials possess the capacity to encapsulate drugs and accurately release them at the target sites (Sattar et al., 2022; Chamundeeswari et al., 2019). Moreover, nanoassemblies based on anti-inflammatory biomolecules possess the capability to reduce inflammation by themselves. Accordingly, the use of high-performance nanomaterials as emerging biomaterials for the treatment of OA is regarded as an urgent strategy.

**FIGURE 1:**
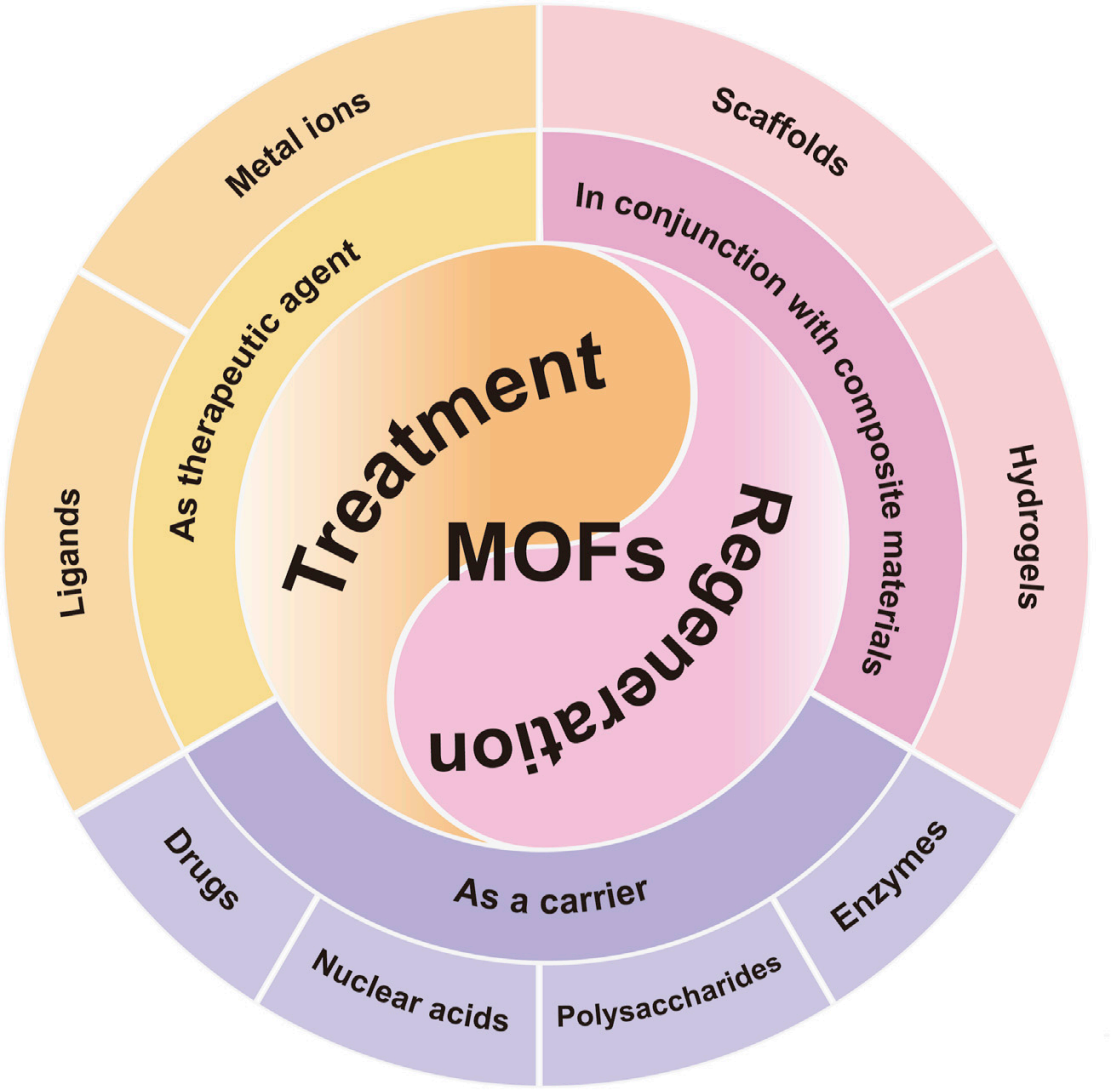
The summary of MOFs and their derivatives in the field of OA treatment. Schematic representation of the three pillars of MOFs application-therapeutic agents, carriers and compositions for scaffold materials.

Metal-organic frameworks (MOFs) are crystalline compounds composed of organic ligands and metal ions. Because of the intrinsic porous properties, thermal and chemical stability, easy surface modification, as well as high biosafety, metal-organic frameworks (MOFs) have been considered as promising nanocarriers in the field of biomedicine (Horcajada et al., 2008; Li L. et al., 2023; Li X. et al., 2023). It is worth noting that a variety of factors including internal stimuli such as redox, pH, and enzymes, as well as external stimuli such as light, temperature, magnetism, ultrasound, and mechanical force have been utilized to trigger the release of guest molecules from MOF-based nanosystems. Meanwhile, these aforementioned nanosystems usually exhibit the ability to react towards multiple stimuli, which can enable them to release chemicals at specified moments or spaces (Gu et al., 2023). More importantly, MOFs and their derivatives with inherent bioactivity can relieve localized inflammation in the treatment of inflammatory diseases (Li et al., 2024; Shyngys et al., 2021). This review article critically summarized the recent advances made in the development of MOFs and their derivatives, as well as their potentials for treating OA. As illustrated in Figure 1, the key emphasis of this review was to discuss the roles of MOFs and their derivatives for the OA treatment and bone regeneration. Moreover, the stability and biological activity of these biomaterials were well evaluated in the above-mentioned OA treatments. Last but not least, this review concluded with an overview of the challenges hindering further development of MOFs and their derivatives and a perspective on the future of the field before their clinical translation.

## 2 MOF as a therapeutic agent

**FIGURE 2:**
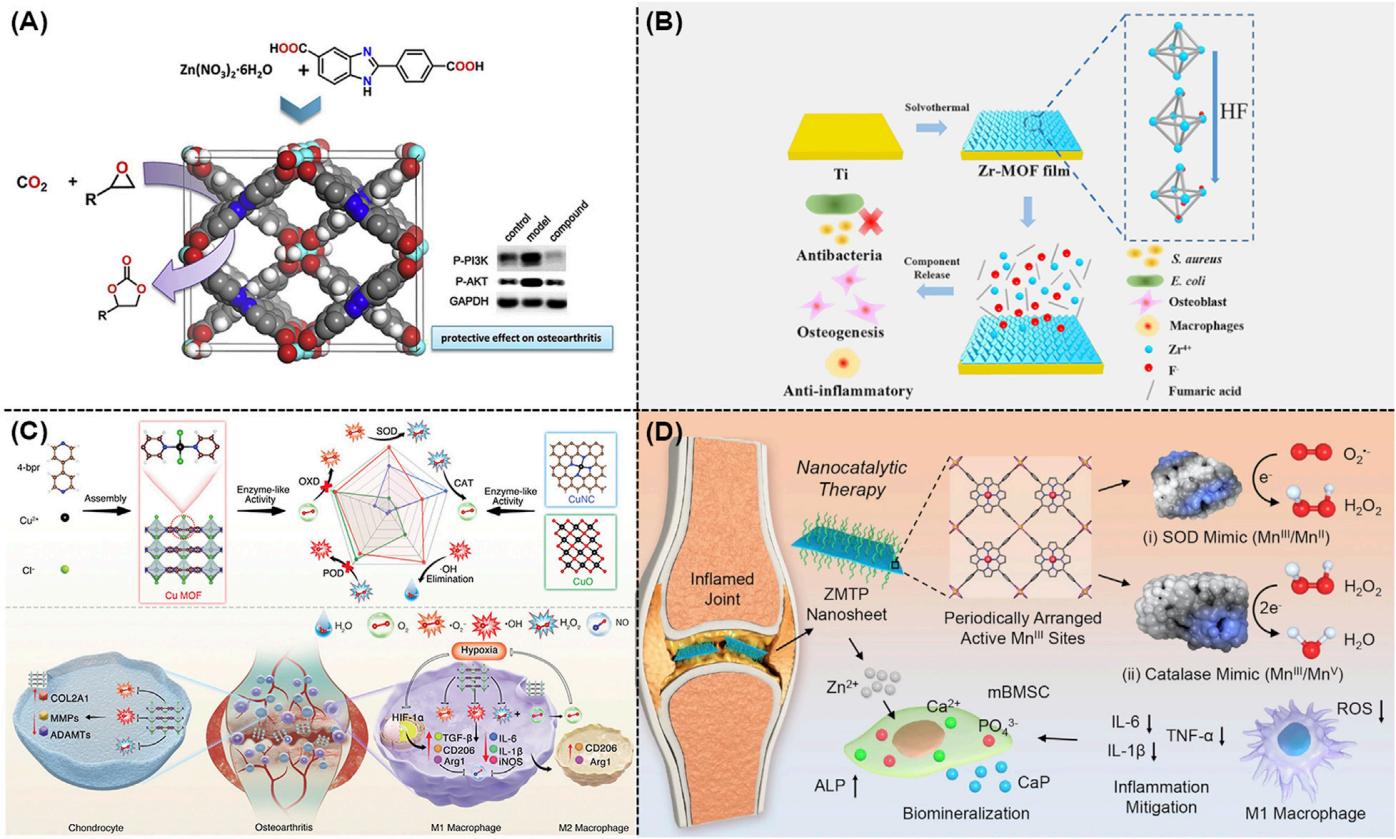
**(A)** Schematic representation of the preparation of Zn(II)-MOF and its effect on activation of the PI3K/AKT signaling pathway (Cai et al., 2020). **(B)** Preparation of Zr-MOF film and its antimicrobial, anti-inflammatory, and osteogenic capabilities (Yan et al., 2022). **(C)** Schematic illustration of Cu-MOF nanozymes for the treatment of OA by scavenging ROS and regulating macrophage phenotype (Yu et al., 2024). **(D)** Schematic illustration of the therapeutic mechanism of ZMTP nanosheets for nanocatalytic treatment of rheumatoid arthritis (Yang B. et al., 2022).

Essential metal ions or clusters including Zn, Mg, Zr, Fe, Cu, and Mn usually held the ability to cure OA, which could also serve as the structure centers of MOFs and their derivatives. As illustrated in Figure 2A, Cheng and co-workers synthesized a novel MOF named as {[Zn(H_2_O)(HL)]·(DMF)_2_(H_2_O)_2_}_n_, which could regulate the inflammation associated with OA via reducing the activation of the PI3K/AKT signaling pathway (Cai et al., 2020; Sun et al., 2020). Tang and co-workers developed a new Mg/HCOOH-MOF, which could serve as a controlled-release drug for treating OA. The magnesium ions derived from Mg/HCOOH-MOF held both anti-inflammatory and osteogenic properties, promising their potentials in the anti-inflammatory therapy and further promotion of osteogenesis (Li et al., 2020b). As shown in Figure 2B, Yan and co-workers prepared a novel Zr-MOF-801 with admirable antimicrobial effect and enhanced osteogenic performance (Yan et al., 2022).

Reactive oxygen species (ROS) could significantly contribute to the development and exacerbation of OA, which emerged as a promising therapeutic target. As a famous nanozyme that came from Fe-based MOF family, Mil-88 with excellent peroxidase activity exhibited the ability to eliminate ROS both *in vitro* and *in vivo*. According to these findings, a very recent study revealed that a feasible Mil-88-based enzymatic healing process could be beneficial for treating OA (Lopez-Cantu et al., 2022). For instance, Mil-88 could enhance the bioactivity of genes related to tissue growth (such as Col-2) and reduce the activity of genes associated with OA tissue breakdown (such as MMP-13), promising its potential to be a novel and advantageous method for treating OA (Hu et al., 2023). Meanwhile, a well-defined Cu-MOF nanozymes were well developed for the treatment of OA using a straightforward self-assembly method, as shown in Figure 2C. According to the density functional theory (DFT), these Cu-MOF nanozymes with excellent superoxide dismutase (SOD) and catalase (CAT) mimicking characteristics had higher antioxidant activity in comparison to other Cu-based antioxidants. As expected, these Cu-MOF nanozymes could alleviate the low oxygen conditions in OA, transform macrophages into the M2 phenotype, prevent cartilage damage, as well as decrease the release of pro-inflammatory factors (Yu et al., 2024). Furthermore, a MOF named as Zn-Mn-TCPP-PVP (ZMTP) were well designed using the coordination effect between the benzoyloxy group of Mn-TCPP and Zn^2+^ (Yang B. et al., 2022). As shown in Figure 2D, these well-developed ZMTP with high SOD activity and CAT activity exhibited great anti-inflammatory and bio-mineralization performances in both *in vitro* and *in vivo* models.

## 3 MOF as a carrier

Considering that there were lots of drugs utilized for treating OA in daily clinic, a variety of carriers were highly required. In comparison to other nanoscale carriers, MOFs and their derivatives held several advantages including a substantial specific surface area for the attachment of cells, a high porosity for the encapsulation of biomolecules, a great feasibility for the surface modification with targeted and responsive molecules, and a high biocompatibility. Therefore, it is suitable for the delivery of various guest molecules, including drugs, proteins, nucleic acids and polysaccharides.

### 3.1 Loading drugs

**FIGURE 3:**
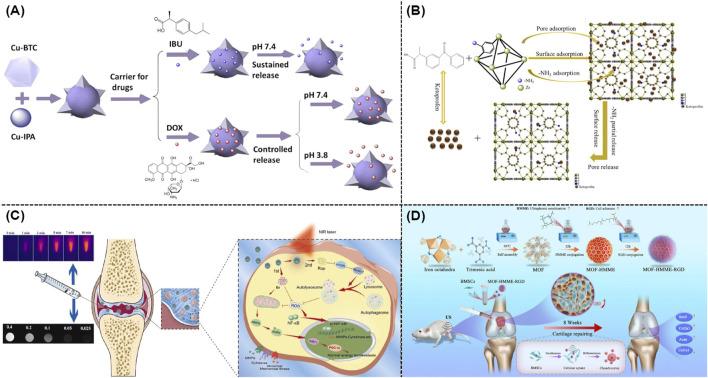
**(A)** Schematic illustration of the application of mixed ligand MOFs as efficient drug carriers for IBU and DOX (Sun et al., 2017). **(B)** Drug loading and release mechanism of UiO-66-NH_2_ as a drug carrier (Li et al., 2019b). **(C)** Mechanisms of dual drug delivery nanoplatforms with cartilage-targeting effect and near-infrared laser response in OA therapy (Xue S. et al., 2021). **(D)** Schematic illustration of the synthesis of MOF-HMME-RGD and relative therapeutic mechanisms (Luo et al., 2024).

According to recent clinical research, the predominant non-steroidal anti-inflammatory drugs (NSAIDs) for the symptomatic management of OA were mainly ibuprofen, ketoprofen, and diclofenac (Richard et al., 2023). Although the localized use of NSAIDs extremely enhanced their absorption, two factors might seriously lead to a decrease in their effectiveness. First, a periarticular microvascular system usually facilitated the rapid drug clearance from joints. Second, the skin played an active role in metabolizing medicines using the cytochrome P450 enzyme. In order to improve the medication usage and relative OA treatment efficacy, well-defined nanoscale carriers were highly needed (Patil et al., 2023). Previous studies demonstrated that MIL-101 (Cr) and MIL-100 could achieve the high loading of ibuprofen and its stimuli-responsive release (Silva et al., 2016). Figure 3A revealed that novel Cu-MOFs composed with multiple ligands were created as efficient carriers for delivering IBU and DOX (Sun et al., 2017). Furthermore, Tan and co-workers utilized zirconium-based MOF (UiO-66) as an efficient drug carrier (Li et al., 2019b). As shown in Figure 3B, by changing different functional groups, these well-developed nano-sized carriers could well response toward different stimulants. As expected, UiO-66-NH_2_ held the best loading and release properties. Furthermore, a MOF-based drug delivery nanosystem based on Sr/PTA-MOF-ketoprofen could reduce inflammation, relieve pain, as well as maintain bone homeostasis (Li et al., 2019a; Li et al., 2020a).

Owing to the acidic nature of joint milieu, the acid-responsive release mechanism was well understood in various MOF-based delivery systems. Itaconic acid-contained ZIF-8 was well prepared as a pH-responsive drug-loaded nanocarrier in which itaconic acid could alleviate OA after the efficient cellular penetration and endocytosis (Yu et al., 2023). Meanwhile, the ZIF-8’s pH-responsive characteristic was utilized to encapsulate neoflavone (NBIF), which could extremely reduce the poor solubility of NBIF and its rapid degradation (Jiang et al., 2023). As shown in Figure 3C, a nano-size hybrid nanocarrier based on mesoporous polydopamine and MOF was rationally developed as an efficient photothermal reagent to promote the OA treatment. The above mentioned nanosystem exhibited rapid responsiveness toward the irradiation of near infrared (NIR) light, leading to the enhanced drug release (Xue S. et al., 2021). Moreover, a zirconium-based MOF was modified with polyphenols and gold, which exhibited excellent photothermal outcomes for the treatment of OA (Xu S. et al., 2023). Recent studies indicated that sonodynamic therapy was utilized in the OA treatment. As shown in Figure 3D, a Fe-based MOF was well conjugated with hematoporphyrin monomethyl ether (HMME) and arginine-aspartate-glycine (RGD) peptides for repairing cartilage defects (Luo et al., 2024). According to the current design, the porous architecture of MOFs could effectively prevent organic acoustic molecules from their self-quenching while ROS could be rapidly generated through the linker-metal charge transfer pathway with high efficacy.

### 3.2 Loading nuclear acids

**FIGURE 4:**
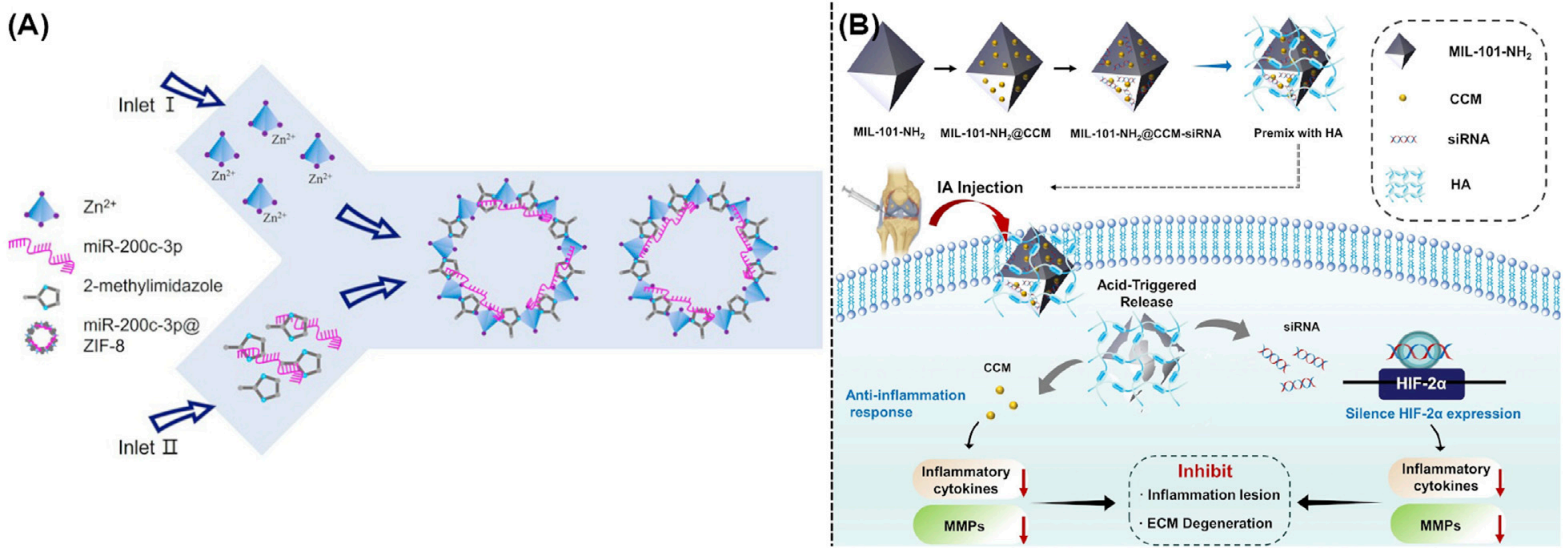
**(A)** Schematic illustration of a one-step microfluidic preparation of miR-200c-3p@ZIF-8 (Yang et al., 2023). **(B)** Synthesis of MIL-101-NH_2_@CCM-siRNA and its usage in the OA treatment (Zhang et al., 2023a).

Recent studies indicated that RNA-based gene therapy was widely utilized to treat OA. This innovative method usually employed microRNAs and anti-miRNAs for polygenic regulation, small interfering RNAs (siRNAs) for gene silencing, and mRNAs for gene supplementation (DeJulius et al., 2024). Although promising, current systemic delivery of RNA usually failed because of the complicated environment of body and the biodegradability of nuclear acids, which also could lead to serious side effects including uncertain retention duration in body, immunological infection, and potential toxicity. Recent studies suggested the applications of MOFs and their derivatives as high-performance nanocarriers for the delivery of nuclear acid (He et al., 2023). The well-defined chemical structures of MOFs and their derivatives endowed them with the possibility of strong interaction with nucleic acid biomolecules, which could occur by encapsulation, physical adsorption, as well as chemical grafting (He et al., 2023). As illustrated in Figure 4A, some researchers utilized ZIF-8 to create miR-200c-3p@ZIF-8 using a facile one-step approach with the assistance of a Y-shape microfluidic chip. This approach allows for the exact manipulation of miR-200c-3p@ZIF-8 with ideal size, resulting in the admirable encapsulation efficacy and transport outcome. As expected, these well-developed miR-200c-3p@ZIF-8 hybrids could significantly lowered the expression of associated inflammatory factors during the OA treatment (Yang et al., 2023). Moreover, siRNA targeting the hypoxia-inducible factor-2α (HIF-2α) gene and the relative anti-inflammation therapy could be well achieved using MIL-101-NH_2_ as efficient nanocarrier. Figure 4B demonstrated that gene and drug loaded MIL-101-NH_2_ could efficiently release siRNA and curcumin in the acidic OA microenvironment, which thus silenced the HIF-2α gene, reduced the levels of pro-inflammatory factors, and avoided the relative cartilage degradation (Zhang et al., 2023a).

### 3.3 Loading polysaccharides

Due to their high biocompatibility and low systemic toxicity, polysaccharides and polysaccharide-based materials were ideal for the treatment of OA. Recent studies demonstrated that polysaccharides could be well utilized as raw materials for the synthesis of ECM, which served as efficient lubricants for lubricating joints. Moreover, polysaccharides could provide a suitable environment for chondrocyte repair, exert antioxidant activity to regulate the OA microenvironment, as well as interact with cell membrane receptors to avoid the attack of inflammatory factors (An et al., 2024; Alcaide-Ruggiero et al., 2023). Although promising, essential strategies were highly required to optimize the current delivery approach and therapeutic efficacy due to the high molecular weight and fragility of various polysaccharides.

**FIGURE 5:**
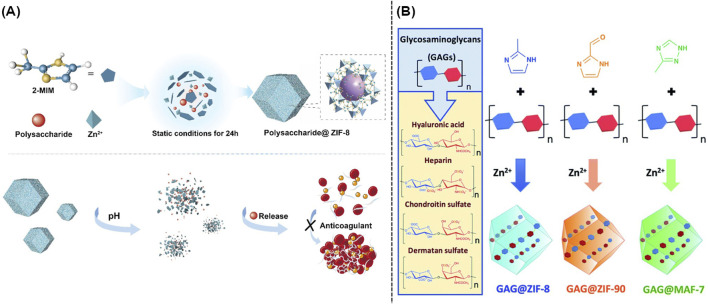
**(A)** Schematic illustration of the synthesis of polysaccharides@ZIF-8 (Zheng et al., 2021). **(B)** One-pot synthesis of GAG@MOFs originated from three different metallic nitrogen heterocyclic frameworks (Velásquez-Hernández et al., 2020).

Traditional carriers including silica, hydrogels, and polymer-based materials were highly limited by their non-biodegradability, low loading efficacy, as well as complicated synthesis. Recent studies indicated that the intrinsic porous structure of MOFs could efficiently adsorb and accommodate polysaccharides. Moreover, polysaccharides could be well loaded into the interior of MOFs via the electrostatic interaction in the mixture containing metal ions, organic ligands, and polysaccharides, which resulted in the direct synthesis of polysaccharide-loaded MOFs (Tong et al., 2021). As shown in Figure 5A, ZIF-8 could encapsulate heparin, fucoidan sulfate, and fucosylated chondroitin sulfate, and hyaluronic acid. The well-developed nanocomposites named as polysaccharide@ZIF-8 followed an acid-responsive property, which could release polysaccharide in the OA microenvironment (Zheng et al., 2021). Furthermore, several pH-responsive MOFs including ZIF-8, ZIF-90, and MAF-7 were rationally designed and utilized to provide the protection for glycosaminoglycans (GAG). As shown in Figure 5B, the above mentioned GAG-loaded nanocomposites could offer new avenues for the further therapy of OA (Velásquez-Hernández et al., 2020).

### 3.4 Loading enzymes

Previous studies demonstrated that natural enzymes or artificial enzymes with antioxidant activity held great potentials in the OA therapy. Accordingly, MOFs were widely utilized to load these enzymes, which could extremely improve their stability and endurance under physiological conditions. For example, platinum (Pt) nanozymes with excellent ROS scavenging and anti-inflammatory activities were encapsulated in ZIF-8 while lantanum (La) as an osteogenic active element was well incorporated via ion exchange, which resulted in the formation of Pt@ZIF-8@La with a bimetallic-organic backbone. Both *in vitro* and *in vivo* results demonstrated that this well-developed multifunctional nanoplatform with high enzyme activity and great ability of ion release exhibited efficient synergistic therapeutic effects of immunomodulation and osteogenesis (Pan et al., 2023). In addition, Pt@PCN222-Mn with multifunctional active sites was involved in the treatment of temporomandibular joint (TMJ) osteoarthritis. This well-prepared nanocomposite could remodel the inflammatory microenvironment of TMJ OA and delay its development through regulating the ROS-NF-κB and p38/MAPK signaling pathways (Zhang et al., 2023).

## 4 MOF as a scaffold material composition

Besides many drugs and biomolecules were widely utilized for the inflammation control and cartilage repairing in clinic, tissue engineering still remained its necessity and specificity in the promotion of new cartilage formation. Combined with the use of MOFs and their derivatives, multifunctional platforms based on scaffolds or hydrogels could result in ideal therapeutic outcomes for OA. The addition of MOFs to scaffolds can endow these scaffold materials with special physicochemical properties and biological effects, so as to better assist the function of scaffold materials.

### 4.1 Scaffolds

**FIGURE 6:**
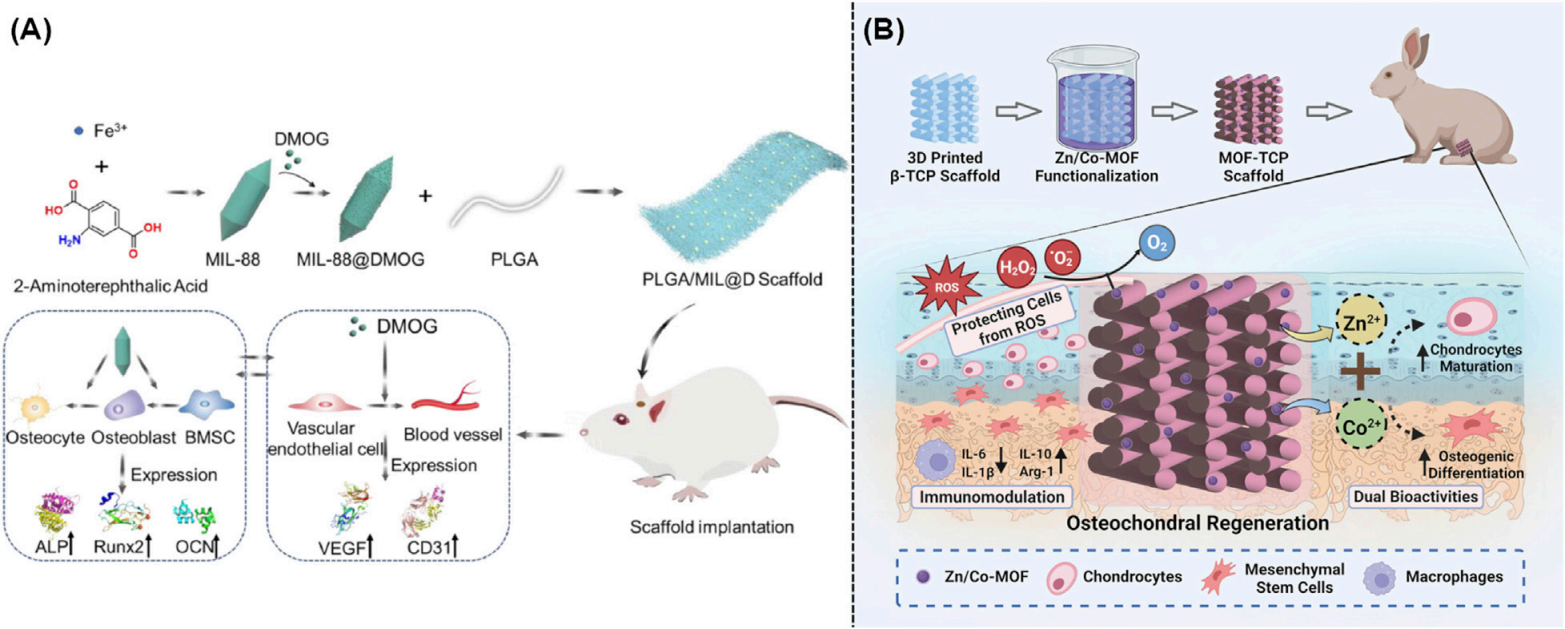
**(A)** Preparation and mechanism of the iron-based MOF-contained hybrid scaffolds for the repair of bone defects (Xu C. et al., 2023). **(B)** Synthesis and mechanism of the MOF-TCP-based scaffold for the treatment of osteoarthritis (Shu et al., 2023).

Recently, approaches including electrostatic spinning, solvent casting, particle leaching, gas foaming and freeze drying, stereolithography, and 3D bioprinting were widely utilized to create artificial scaffolding (Wu et al., 2022). MOFs and other biomaterials were well integrated with the above mentioned artificial scaffolding to develop novel hybrid scaffolds for the OA therapy. As expected, these well-designed multifunctional platforms exhibited excellent anti-inflammatory and antioxidant activity, which could result in the ideal cartilage repairing. For example, poly(lactide-co-glycolide) (PLGA) and dimethyloxallyl glycine (DMOG) loaded iron-based MIL-88 were utilized to prepare novel nanofibrous scaffolds via electrostatic spinning technology, which could serve as artificial extracellular matrix for the promotion of osteogenesis and angiogenesis (Wu et al., 2022). Figure 6A illustrated the synthesis approach of hybrid scaffold and their mechanism in the OA treatment. Compared with the scaffold without any modification, the electrostatically spun mats became rougher upon the addition of MOFs, which thus made the gradual breakdown and absorption of hybrid scaffolds more easily and allowed for the ideal release of iron ions within the injury sites (Xu C. et al., 2023). Furthermore, another novel bioceramic scaffolds with good mechanical characteristics and bioactivity were involved into the field of bone tissue engineering. As well known, β-tricalcium phosphate (β-TCP) was widely utilized for the bone regeneration because it could gradually degrade into calcium and phosphate ions upon the acid microenvironment. Figure 6B illustrated that the addition of Zn/Co-MOF into β-TCP could result in the formation of a novel hybrid scaffold, which could reduce localized inflammation and repair osteochondral tissue through the elimination of ROS and release of ions (Shu et al., 2023).

Electrospun fibrous membrane was a novel bionic flexible scaffold that could be utilized to repair tissue injuries, particularly gradient tissue damage that occurred at the interfaces of tendons and bones. Recently, a metal-organic framework-based bipolar metal flexible electrospun fibrous membrane was prepared as a new kind of biomimetic flexible scaffold with multiple layers using electrostatic spinning technology. If the fibers degraded, metal ions such as Zn^2+^ and Cu^2+^ might be released, which could regulate collagen formation, osteoclast differentiation, and angiogenesis (Yang R. et al., 2022). In addition, researchers also created an asymmetric bilayer PCL/Col membrane that was changed in the same way by ZIF-8 crystals. The composite membrane made of PCL/Col/ZIF-8 included two layers including a barrier layer and a crystal layer. Several admirable properties including increased tensile strength, low pH responsiveness, and controlled degradation rate endowed these well-developed composites with great performance in the bone regeneration both *in vitro* and *in vivo* (Xue Y. et al., 2021).

### 4.2 Hydrogels

**FIGURE 7:**
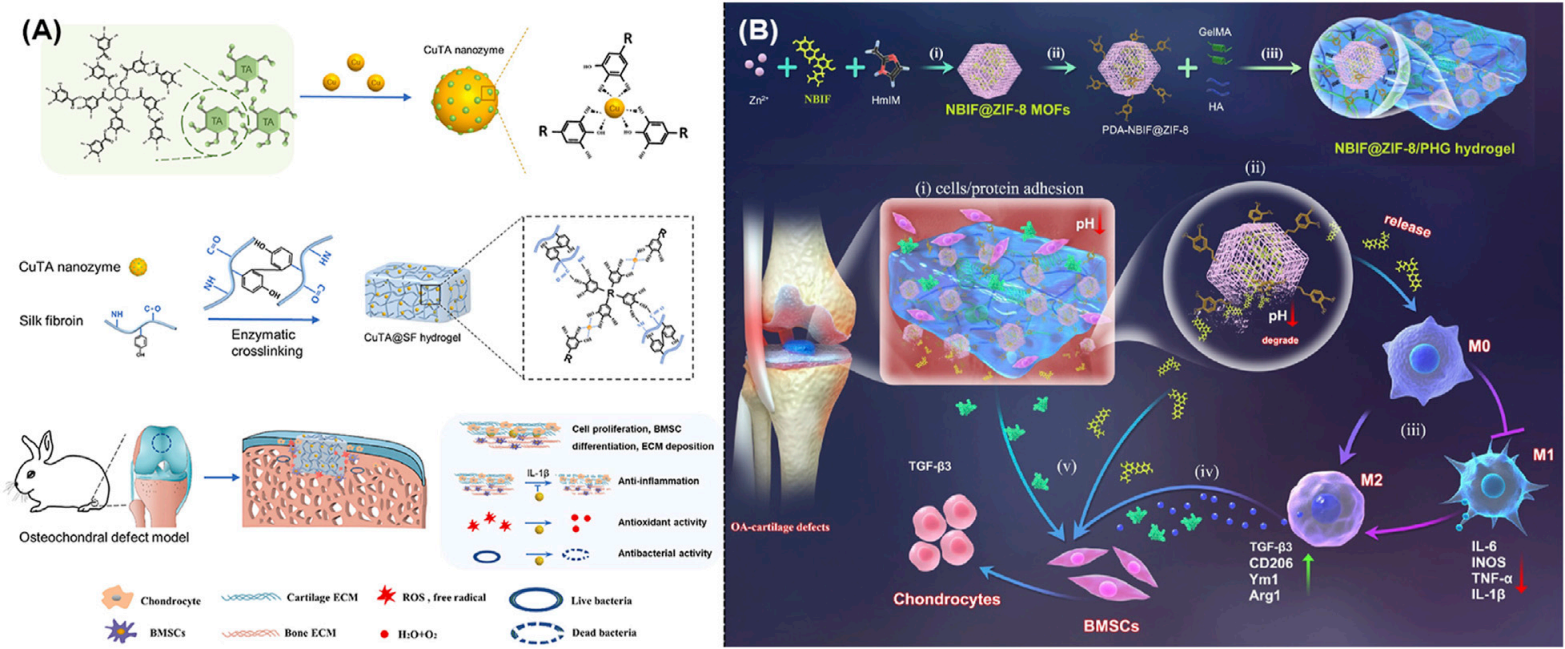
**(A)** Schematic illustration of the synthesis of CuTA nanozymes and CuTA@SF hydrogels, as well as their application in the repair of bone defects (Cao et al., 2023). **(B)** Schematic illustration of the design and usage of a MOF-based hydrogel to promote OA-induced cartilage defect repair (Jiang et al., 2023).

It was well known that hydrogel held a striking structural similarity to the ECM of bone and cartilage, which could gradually build up a three-dimensional structure, well integrate with the surrounding tissues, and provide essential support and protection during the tissue repairing. Recent studies indicated that hydrogels held the ability to encapsulate medicines or growth factors that aided in the cartilage healing. As expected, Figure 7A illustrated the creation of CuTA@SF using MOFs, which could speed up the cell proliferation and extremely enhance the tissue regeneration (Cao et al., 2023). Meanwhile, IA-ZIF-8@HMs was effectively prepared using a one-step microfluidic approach. During the treatment of OA, IA-ZIF-8 could be released from hydrogel microspheres (HMs), further decreasing the localized inflammation (Yu et al., 2023). Moreover, researchers designed and created a hybrid hydrogel (G-GH/CL-CD-MOF) by mixing gelatin-glucosamine hydrochloride with cross-linked cyclodextrin MOF. The optimum porosity structure of this drug delivery system endowed it with a long-term, sustained release of ibuprofen and excellent anti-inflammation treatment of OA (Yang H. et al., 2022). On the other hand, researchers also mixed polycaprolactone and gelatin mats with different amounts of glucosamine loaded ZIF-8 nanoparticles with the ideal goal to achieve the growth of cartilage tissue (Ranjbar et al., 2023). As shown in Figure 7B, novel NBIF@ZIF-8/PHG hydrogels with robust mechanical characteristics and the ability to recover from deformation were well created as efficient alternatives in the cartilage repairing (Jiang et al., 2023). In addition, the tendon-targeting peptide and cartilage-targeting peptide were well loaded in ZIF-8 and Mg-MOF, respectively. Subsequently, these peptide-loaded MOFs were immobilized in methacrylate gelatin using UV-induced photopolymerization to produce LZIF-8/WMg-MOF@GEL, which could achieve a tailored ion delivery platform to both tendon and cartilage (Ma et al., 2024).

## 5 Conclusion and perspective

With the rapid development of medicine and material science, the use of smart MOFs and their derivatives as emerging biomaterials for the OA treatment has been one of the most important topic of biomedical engineering. In this review, we summarized the latest studies focused on the use of MOFs and their derivatives as ideal carriers for drugs and biomolecules or main composites of hybrid scaffolds and hydrogels in the treatment of OA. The greatest advantage of these well-designed MOFs and their derivatives for OA therapy were concluded as that they could significantly overcome some limitations of drugs and biomolecules such as poor solubility, short retention time, poor targeting capacity, and ease of biodegradability. Moreover, the essential role of MOFs and their derivatives in tissue engineering was well understood after that they were integrated with scaffolds and hydrogels to construct hybrid platforms. Although these MOFs held promising clinical potentials, their further clarification was still necessary on some certain issues. First, there was still a lack of knowledge regarding the drug delivery and release dynamics in MOFs. Second, the *in vivo* stability, organ distribution and metabolites of MOFs remain unknown, limiting further development and utilization. Third, how to promote tissue regeneration rather than just control inflammation, and the function of MOFs in this process need to be further explored. Accordingly, this review not only emphasizes the considerable potentials of MOFs and their derivatives in the OA treatment, but also anticipated an increase in the number of relevant studies applying MOFs to the treatment of OA in the future. Last but not least, we believed that it would be a transformative clinical achievement by using MOFs as efficient biomaterials to promote the treatment of OA and its related diseases.
